# Temporal bone pneumatization: A scoping review on the growth and size of mastoid air cell system with age

**DOI:** 10.1371/journal.pone.0269360

**Published:** 2022-06-03

**Authors:** Okikioluwa Stephen Aladeyelu, Kehinde Samuel Olaniyi, Samuel Oluwaseun Olojede, Wonder-Boy Eumane Mbatha, Andile Lindokuhle Sibiya, Carmen Olivia Rennie

**Affiliations:** 1 Discipline of Clinical Anatomy, School of Laboratory Medicine and Medical Sciences, Nelson R. Mandela School of Medicine Campus, University of Kwazulu-Natal, Durban, South Africa; 2 Nelson R Mandela School of Medicine, School of Laboratory Medicine and Medical Sciences, University of Kwazulu-Natal, Durban, South Africa; 3 Lake, Smit & Partners Inc., Durban, South Africa; 4 Department of Radiology, Inkosi Albert Luthuli Central Hospital, Durban, South Africa; 5 Discipline of Otorhinolaryngology-Head and Neck Surgery, School of Clinical Medicine, Nelson R. Mandela School of Medicine Campus, University of Kwazulu-Natal, Durban, South Africa; 6 Department of ENT, Inkosi Albert Luthuli Central Hospital, Durban, South Africa; Flinders University, AUSTRALIA

## Abstract

The interest in the mastoid air cell system arose from the association between temporal bone aeration and otitis media. Its size and growth have been considered when planning chronic and middle ear surgeries. The objective of this review was to explore the literature on the size of mastoid air cells with age, highlighting various growth rates reported and mapping out areas yet to be fully understood for further research. A three-step systematic search was conducted for available literature on the subject matter viz; Google Scholar, Medline, Cochrane Library, and PubMed. Eligibility criteria guided the study selection, and eligible studies were subjected to appraisal using screening and quantitative criteria of mixed-method appraisal tool. A data extraction form was developed to extract information from eligible studies. Nine studies met the eligibility criteria. 55.6% of the included studies were conducted among the east and south Asian population, 33.3% were conducted among Scandinavians, and 11.1% in South America. Age groupings varied among studies; 33.3% utilized 1-year age grouping, 33.3% utilized 5-year age grouping, 11.1% utilized 10-year age grouping. In reporting the size of mastoid air cells across age groupings, 66.7% utilized area, 22.2% utilized volume, while 11.1% utilized both area and volume. Findings from this review showed that the mastoid air cells’ size with respect to age differs among populations of different origins. The most common measurements were the area of air cells. The highest growth rate was reported up to 30 years. Findings also show the influence of sex on the size of mastoid air cells and growth rate with age, as females were reported to have larger air cells with rapid growth until puberty. However, the male mastoid air cell system continues a steady growth after puberty and becomes larger. Information still lacks in the volume of air cells in pediatric pneumatization.

## Introduction

The temporal bone, a pair of bones located on the lateral skull that could be ignored because of its size but its anatomical complexity, could pose a challenge in interpreting anatomical details and diagnosis of various pathological conditions [1]. Each temporal bone consists of four parts: a squamous part, which is the antero-superior part of the bone; a tympanic part, which is a curved plate below the squamous part and anterior to the mastoid process; a styloid process, which is a slender and pointed bone which projects antero-inferiorly from the inferior aspect of the temporal bone; and a petromastoid part, which is relatively large consisting of the petrous and mastoid process [2, 3]. The petromastoid part houses the ear components, contains numerous openings and canals through which structures enter and exit the cranial cavity, and comprises a compact bone and trabeculae that are variably pneumatized [2, 4, 5].

Pneumatization is the presence or development of air-filled cavities or epithelial-lined air cells within cranial components that remain after the pneumatization process [6]. The pneumatization of temporal bone serves as a prognostic factor in middle ear surgery and could be considered once related surgeries are planned [7–9]. Temporal bone pneumatization has also been reported to give a protective function, acting as a shock absorber in patients with lateral skull-based fracture and traumatic brain injury [10, 11], which have had a devastating impact on many individuals, approximately 69 million individuals worldwide and affect a significant number of people in South Africa [12, 13]. However, minimal pneumatization of the temporal bone is a characteristic of otitis media [14, 15], which is one of the global burdens of disease [16–20], and significantly high in South Africa among younger children and older children with prevalence of 31.4% and 16.7% respectively [21, 22].

Temporal bone pneumatization is a process that begins during prenatal development (the mastoid antrum), achieved by transitioning the temporal bone into air cells which were first described by Schwartze and Eysell in 1873 and completed by Zuckerkandl in 1879 [9, 15, 23–25]. At about the 24^th^ week of intrauterine life, the mastoid antrum is the only visible air cell, lined by a single flat layer of epithelium distinguished from bone by subepithelial connective tissue, which activity is largely responsible for air-cell formation [6, 26, 27]. The development of mastoid air cells (also called mastoid cells of Lenoir or air cells of Lenoir) is preceded by the formation of bone cavities that contain primitive bone marrow, which dedifferentiates into a loose mesenchymal connective tissue [23, 24, 28]. Invagination of the epithelial mucous membrane is followed by atrophy, leaving a thin residual lining membrane attached to the periosteum. “Retraction” of the lining membrane and the subepithelial bone resorption further enlarges air cells [24, 26, 28, 29].

After birth, the mastoid air cells are readily visible as hollowed-out spaces lined by flattened, non-ciliated squamous epithelium [30]. These air cells exhibit variability in size and extent [31]. At the superior and anterior parts of the mastoid process, air cells are large and irregular and contain air. Towards the inferior part of the process, they diminish in size, while those at the apex of the process are frequently quite small and contain marrow [27, 31–34]. As growth continues, mastoid air cells communicate with the middle ear via the mastoid antrum and the aditus ad antrum and extend variably to petrous parts and around the inner ear [27, 31, 35]. Air cells may also infiltrate the zygomatic, the squamous, the styloid, and the occipital regions, resulting in accessory pneumatization [34, 36]. Imperatively, there is a gradual reduction in air cells throughout life with additional loss in the elderly both at the base and more reduction at the apex [37].

Exposure of the middle ear to infections such as otitis media could affect the size of the mastoid pneumatization [15, 38]. This is because the mastoid air cell system development has been considered to be impeded by repeated and prolonged otitis media [39, 40]. Other factors that could influence the mastoid air cell system include; genetics, environment, nutrition, and diseases [6, 9, 30]. Therefore, knowledge of the size of mastoid air cells with age is beneficial to researchers in Otorhinolaryngology and Clinical Anatomy.

In the past decades, various studies on temporal bone pneumatization and mastoid air cells have been conducted [6, 41]. However, very few have reported the size of the mastoid air cells concerning age. Therefore, this review is undertaken to explore available age-related studies on the size of the mastoid air cells, highlighting growth rate and size in terms of area and volume, and identifying possible variations regarding ethnicity and sex.

## Methods

This is a scoping review of the available literature on the growth and development of the mastoid air cell system. This review employed the framework outlined by Arksey and O’Malley [42], with further recommendations by Levac and colleagues [43]. These include: identifying the research question; identifying relevant studies; study selection; charting the data; and collating, summarizing, and reporting the results.

### Research question

The research question guiding this review was: “what are the age-related studies that have reported the size of the mastoid air cells in areas and volumes”? Two sub-questions were further used: (i) In which population and countries were these reported? (ii) What age range or groupings were used in reporting these studies?

### Search strategies

A three-step systematic search of the literature was conducted on Google Scholar (Google Inc, Mountain View, California), PubMed and Medline (National Centre of Biotechnology Information, Bethesda, Maryland, United States), and Cochrane library (Access provided by University of KwaZulu-Natal Libraries) electronic databases. The keywords used for this search include temporal bone, pneumatization, mastoid bone, mastoid air cells, mastoid pneumatization, air cell growth, air cell size, and mastoid aeration. These keywords were used alone and in combination with Boolean operators (OR, AND) such as “temporal bone AND pneumatization” OR “mastoid bone AND mastoid air cells” OR “mastoid pneumatization AND air cell size” OR “Temporal bone AND air cell growth” OR “mastoid bone and mastoid aeration”.

The first step search made use of keywords separately and in combination using Google Scholar and PubMed databases only. This was followed by analyzing the text words in the titles and abstracts of the retrieved papers and index terms used to describe the articles. A second search using identified keywords and index terms were used across all databases. The third step search was also conducted across all databases using a reference list of all identified articles.

### Inclusion criteria

The following inclusion criteria were adopted during the literature search: All research and review articles, reports, and books available on temporal bone anatomy and its pneumatization; Research and review articles on mastoid air cell system, growth of air cells, and size documented between 1940–2021; Articles, reports and books available in English Language, articles already translated to the English language and articles that were available in dual languages.

### Exclusion criteria

Research articles, reviews, and reports on temporal bone pneumatization and mastoid air cells before 1940 were excluded during study selection (age-related study on mastoid air cell systems was first reported in 1940 by Diamant). Studies on the classification of temporal bone pneumatization, pathology, and non-age-related studies on mastoid air cell systems were also excluded.

### Study selection

A set of questions in line with the study’s objective was used to assess the relevance of studies identified during the literature search. Study selection was done by two authors (OA and KO) who screened titles and abstracts of all retrieved studies to assess eligibility. When eligibility could not be determined, full articles were retrieved.

### Quality appraisal

The quality appraisal of the eligible studies was conducted using screening criteria for all types of study and quantitative descriptive criteria of the Mixed Methods Appraisal Tool (MMAT)- Version 2018 [43]. This was used because this review involves studies with measurements. OS, KO & SO designed a form to assess the quality of eligible studies. The form consists of two screening questions for all study types and five criteria that apply to quantitative descriptive studies [Table 1]. CR, AS & WM reviewed this form. The appraisal was done by SL and OF, who are not part of this review authorship, to avoid bias. A score of 20% is given when an eligible study fulfills one quantitative criterion, 40% if it fulfills two criteria, 60% if it fulfills three criteria, 80% if it fulfills four criteria, and 100% if it fulfills all quantitative criteria.

**Table 1:** Mixed Methods Appraisal Tool (MMAT), version 2018 indicators for screening questions and quantitative descriptive studies (adapted from Hong *et al*. [44]).

Category of study designs	Methodological quality criteria
Screening questions	*1*. *Are there clear research questions or objectives*?
	*2*. *Do the collected data allow to address the research questions*?
Quantitative descriptive studies	*1*. *The sampling strategy is relevant to address the research questions*
	*2*. *The sample is a representative of the population study*
	*3*. *Appropriate and validated measurements*
	*4*. *Low risk of nonresponse bias*
	*5*. *Appropriate statistical analysis was done to answer the research question*

**Note:** Studies were of acceptable quality when the first two screening questions for all study types and at least one of the indicators in the qualitative criteria were met.

### Data extraction and analysis

A form was developed as a data extraction instrument by three authors (OA, SO) to extract key information such as author, date, title, the aim of the study, method, population, country of study, age range, age grouping, sample size, most significant outcome, and other important outcomes. The extraction form was subjected to review. CR and AS independently used this form to extract data from all eligible studies.

All data were compiled on a spreadsheet and imported into Microsoft Excel 2010. In addition, the content analysis of each paper included in this review was done, and a line graph was used to present the pattern and growth rate of mastoid air cells.

## Results

### Description of included studies

**Fig 1:**
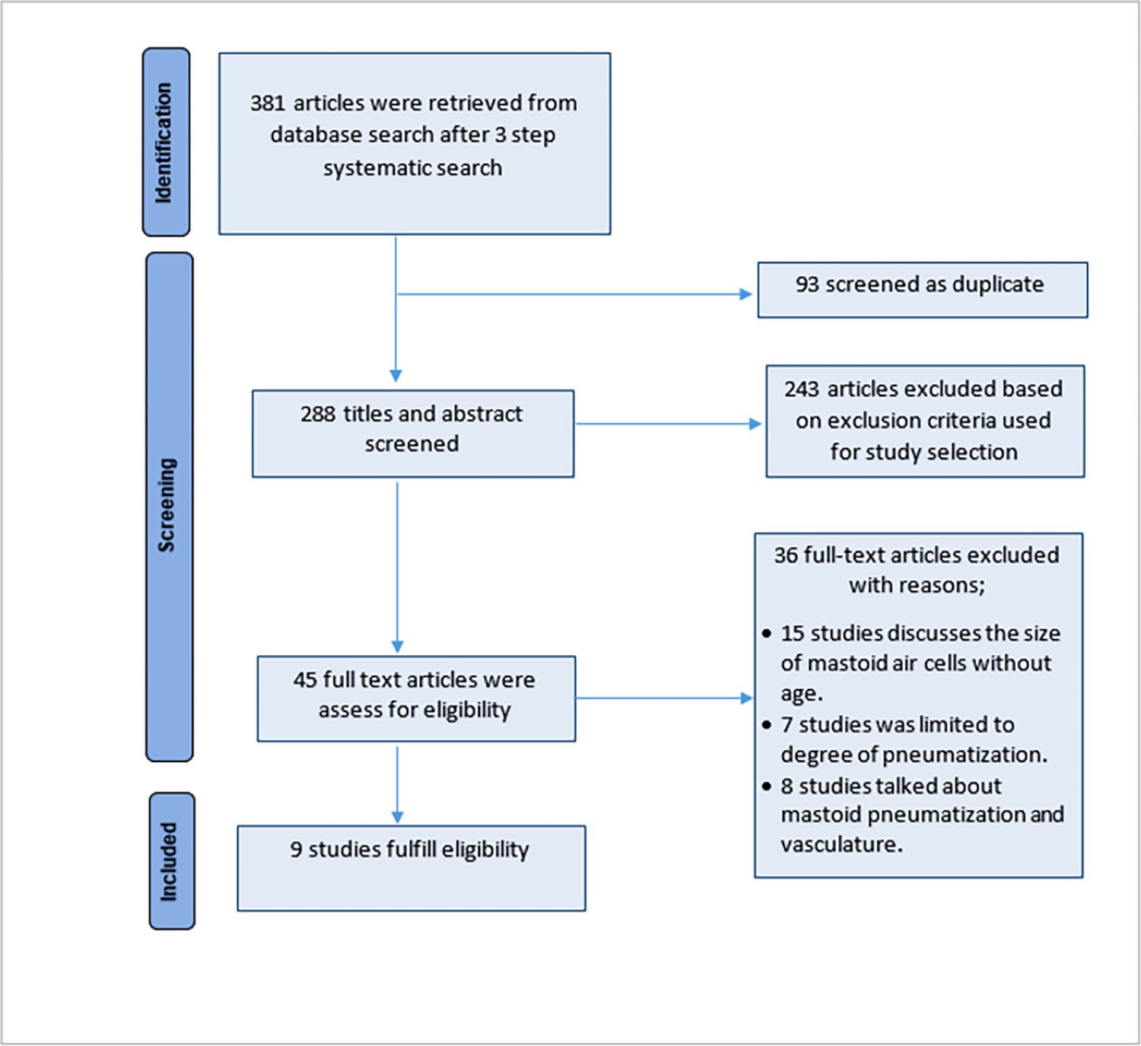
Flow diagram for study selection.

A total of 381 articles were identified during the literature search, including research papers, reviews, reports, and books. Ninety-three duplicates were removed. After screening titles and abstracts, 243 articles were excluded based on the following exclusion criteria; studies reported before 1940, studies reporting petrous pneumatization, studies reporting classification of temporal bone pneumatization, studies reporting pattern of pneumatization, and pathological-related studies. Finally, 45 full-text articles were reviewed for eligibility. Of them, 36 were excluded because they were non-aged related studies on the size of mastoid air cells and limited to degree of mastoid pneumatization. However, only 9 studies were considered to be eligible and included in this review (Fig 1).

**Fig 2:**
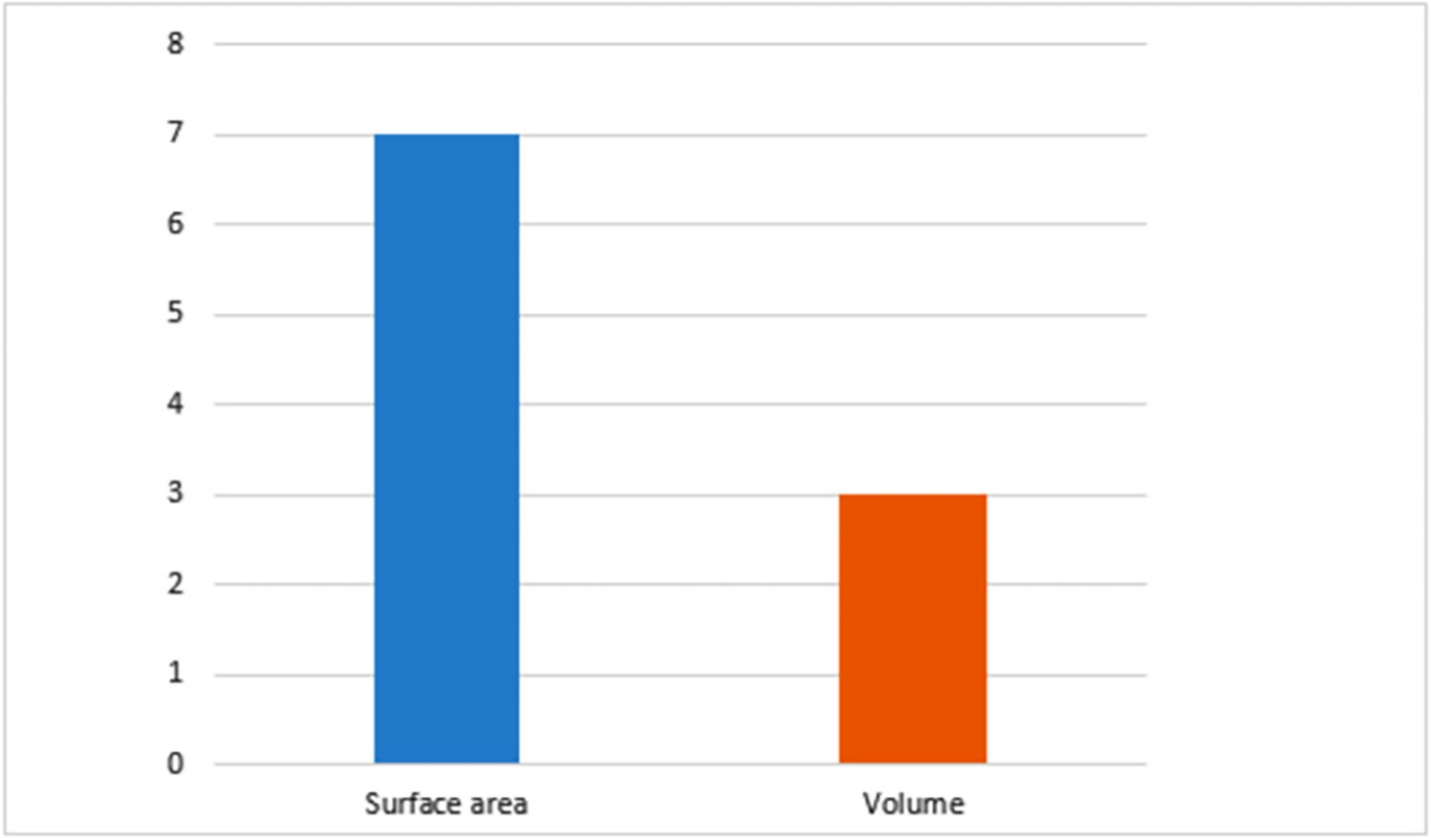
Bar chart showing the frequency of measurement methods of the size of mastoid air cells with age in the literature from 1940 till present. The X-axis displays measurements of air cells, and the Y-axis illustrates the number of articles.

**Fig 3:**
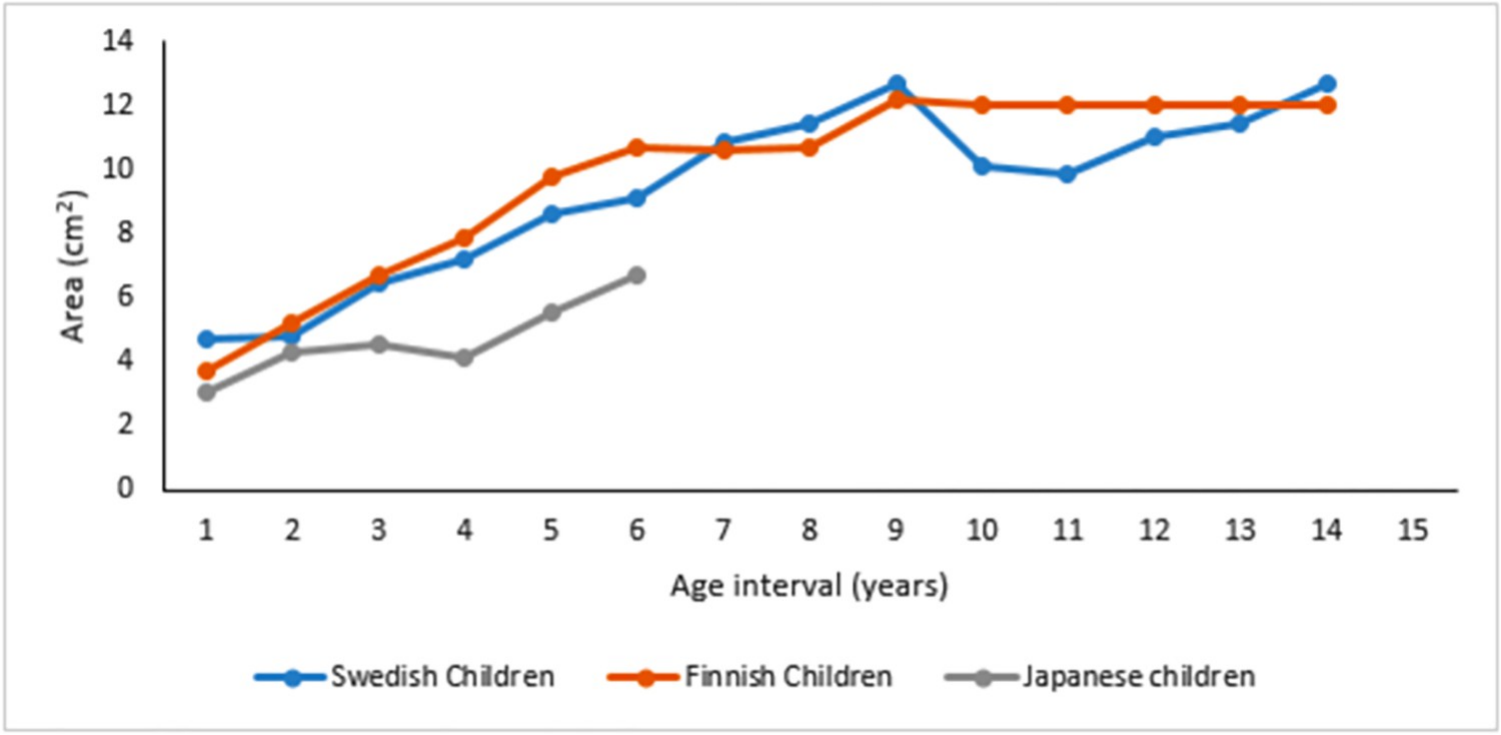
Line graph showing growth rate and size of mastoid air cell systems of studies that utilized 1 year age grouping. X-axis displays age in years, and Y-.axis illustrates the size of the mastoid air cells.

**Fig 4:**
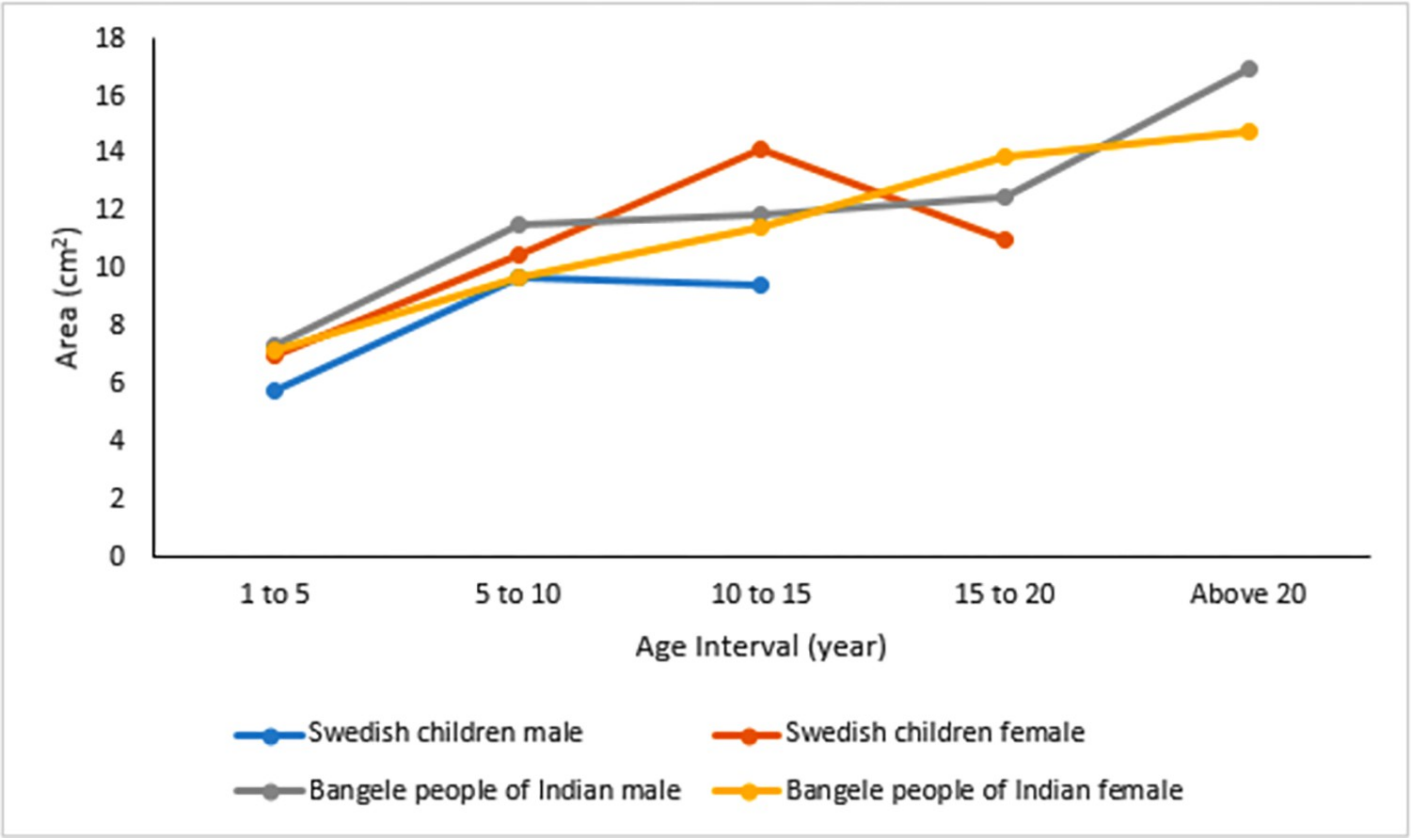
Line graph showing growth rate and size of mastoid air cell systems of studies that utilized 5 year age grouping. X-axis displays the age range in years, and Y-.axis illustrates the size of the mastoid air cells.

**Fig 5:**
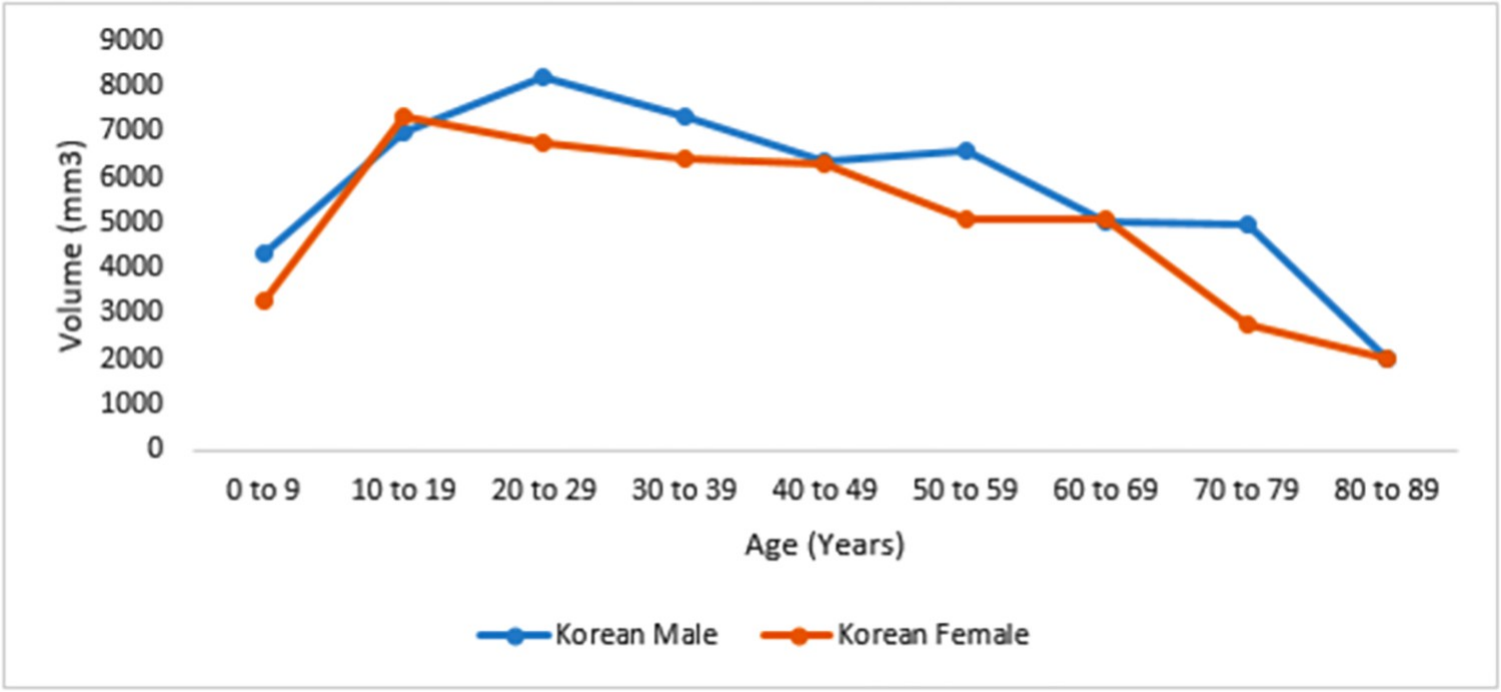
Line graph showing growth rate and size of mastoid air cell systems of study that utilized 10 year age grouping. X-axis displays age range in years, and Y-.axis illustrates the volume of the mastoid air cells.

Table 2 summarizes the included studies and their major findings, while Fig 2 shows the level of knowledge regarding the area and volume of air cells with age. Figs 3–5 show graphical representations (line graph) of patterns and growth rate of mastoid air cells regarding ethnicity and/or sex. The majority (55.6%) of the included studies were conducted among east and south Asian population [45–49], 33.3% were conducted among Scandinavians (North Europe) [41, 50, 51], while 11.1% in South America [4]. The majority (55.6%) of the included studies were conducted in high-income countries, while 44.4% were conducted in middle-income countries (upper & lower). The largest sample size used is 430 [50], and the lowest is 28 [6].

**Table 2:** Table of characteristics of included studies.

Author (Date)	Country (Population)	Sample Size	Male (%)	Female (%)	Age	Aim of Study	Methodology	Major findings mastoid air cell growth	MMAT Score
Diamant, 1940	Sweden (Swedish Children; pre-antibiotic era)	180	43.9%	56.1%	1 to 15 years	To systematically study the mastoid air cells (otitis media and pneumatization of mastoid bone)	A prospective study from 1932 to 1938. Measurement was done by planimetry from lateral X-ray films. Age groupings: 5 years	• This prospective study found out that the area of mastoid air cells increases with age and terminates at puberty. • Mastoid Pneumatization in females was larger and increased significantly. • The mean size reported: 12.04 ± 0.37 cm^2^	60%
Kawamura *et al*. 1963^*b*^	Japan (Japanese Children)	116	-	-	1–6 years	To study the normal development of the mastoid pneumatic cells	Lateral mastoid X-ray film measurements. Age grouping: 1 year	• A linear pattern of air cell growth was observed from age 1–6 years. • The mean size reported: 4.71 cm^2^	60%
Rubensohn, 1965	Sweden (Swedish Children)	430	-	-	1–15 years	To investigate mastoid pneumatization in children at various ages.	Mastoid X-ray measurements. Age grouping: 1 year	• The mastoid air cells system continues to enlarge throughout the years, 1 to 15 years. • A linear pattern of air cell growth was observed from age 1–6 years. • The mean size reported: 9.32 cm^2^	80%
Arora *et al*., 1973	Indian (North Indian Children)	100	50%	50%	5 to 15 years	To measure mastoid Pneumatization in children	Roentgenographic planimetric measurements of North Indian skull. Age groupings: 5 years	• Area of mastoid Pneumatization increases with age by 2 cm^2^ every 5 years, with females having larger pneumatization than males. • Pneumatization terminates in puberty. • The mean size reported: 9.02 cm^2^	60%
Qvarnberg, 1981^*b*^	Finland (Finish Children)	232	-	-	Birth to 16 years	Mastoid air cell system and otitis media	Planimetric measurement of X-rays Age grouping: 1 year	• The mastoid air cells system continues to enlarge throughout the years, 1 to 15 years. • A linear pattern of air cell growth was observed from age 1–6 years. • The mean size reported: 9.66 cm^2^	80%
Chatterjee *et al*., 1990	Indian (West Bengal: Bengalee People)	100	50%	50%	6 months to 60 years	To measure the size of mastoid air cell system in normal subjects in various age groups or either sex and compare with findings with those reported in other countries	A Cross-sectional study using 2D radiographic planimetric measurements of lateral X-ray films. Age groupings: 5 years	• The mastoid air cell system development continues even after 20 years of age as mastoid air cells increase for both sexes, but males have larger pneumatization than females. • Mean size reported: • 12.05 ± 0.67 cm^2^ (male) • 11.45 ± 0.70 cm^2^ (female)	60%
Isono *et al*. 2003	Japan	80	53.75%	46.25%	1–18 years	To describe the measurement of infants’ mastoid air cell system and its developmental changes with age.	Volume measurement of High-Resolution Computed Tomography (CT) images of 2mm slice thickness. Age grouping: Not specified	• It was confirmed that the mastoid air volume increased with age. • By 9–10 years old, it had reached about 80% of the volume of an adult, • At late puberty (14–15 years), it had reached mean adult value. • The mean size reported: 5.97 ml	60%
Lee *et al*., 2005	Korea (Korean population)	102	49%	51%	6–84 years	To report age-related variation of mastoid Pneumatization with the application of 3D computer-based volume measurement.	A retrospective study using 3D-MPVR on CT images of 2.5 mm slice thickness. Age grouping: 10 years	• Volumetric measurements of mastoid air cells revealed that mastoid aeration continues to grow until the 3rd decade of life, and a decline in growth occurs thereafter. • Mean size reported • 3813.85 mm^3^ (Children: 0-10yrs) • 7095.20 mm^3^ (Adult: 19-44yrs)	60%
Hill, 2011	Columbia, Missouri (Human Cadaveric Temporal Bone)	28	-	-	0–25 years	To systematically quantify the normal development of pneumatized spaces. (Ontogenetic study)	A cross-sectional sampling of 28 human temporal bones using 3D reconstruction Quant3D Software and method of ROI and VOI Age grouping: 5 categories (infant, young child, middle child, adolescent, & adult)	• Temporal bone is not well-pneumatized in infants as it is limited to the mastoid antrum • Surface area and volumes of pneumatized spaces double before age 4 • There is a twofold to threefold increase in air cells’ size between age nine and adulthood.	60%

^***b***^ Retrieved from the review and reference study on the growth rate and size of the mastoid air cell system and mastoid bone (Cinamon, 2009).

All included studies were retrospective studies [6, 45–51], except for Diamant [41] which was a prospective study. Age groupings varied among studies; 33.3% utilized 1 year age grouping [45, 50, 51], 33.3% utilized 5 years age grouping [41, 46, 47], 11.1% utilized 10 year age grouping [49], 11.1% utilized inconsistent age grouping [6], while 11.1% age grouping was not defined [48]. Most (55.6%) of the studies included sex in their study [41, 46–49]. In reporting the size of mastoid air cells across age and age groupings, the majority (66.7%) utilized area [41, 45–47, 50, 51], 22.2% used volume [48, 49], while 11.1% used both area and volume [6].

### Methodological quality of the eligible study

The nine included studies were deemed of very good quality as they answered the first two screening questions and fulfilled at least three quantitative criteria of MMAT. On the quantitative criteria, 7 met three criteria- 60% while two met four criteria- 80% (Table 2).

### Mastoid air cell growth rate reported in studies of 1 year consecutive age grouping in area

Three of the included studies that utilized 1-year age grouping were conducted among Swedish, Finnish, and Japanese children [46, 50, 51]. A linear pattern of air cell growth was observed from age 1–6 years, and the mastoid air cells system continues to enlarge throughout the years, 1 to 15 years, except for the study among Japanese children, which was limited to age 6 (Fig 3).

### Mastoid air cell growth rate reported in studies of 5 years consecutive age grouping in area

Three of the included studies utilized 5-year age grouping [41, 46, 47], but only the study of Diamant [41] and Chatterjee *et al*. [47] among Swedish children and Bangele people of Indian gave information from age one. Fig 4 showed rapid growth patterns of mastoid air cell systems for both studies among males and females. Among Swedish children, the area of mastoid air cells increases with age by about 4 cm^2^ in every 5 years but terminates at puberty, 10 years for males and 15 years for females. Mastoid air cells in females was larger and increased significantly. However, among Bangele people of Indian, mastoid air cells increase by about 2 cm^2^ every 5 years, and it continues even after 20 years of age for both sexes, both at a slower rate with males having larger pneumatization than females.

### Mastoid air cell growth rate reported in studies of 10 years consecutive age grouping in volume

Only one study in the included studies utilized 10-year age grouping utilized volumetric measurements of mastoid air cells among the Korean population [49]. Fig 4 showed that mastoid aeration grows and increases at a faster rate from birth to the early 2^nd^ decade of life. Between the late 2^nd^ and 3^rd^ decades of life, the air cells continue to grow slowly. It slowly declines after the 3rd decade of life, then rapidly after the 7^th^ decade of life. Among females, the volume of air cells increases rapidly but experiences an earlier slow growth rate (Fig 5).

## Discussion

Temporal bone pneumatization is a process that begins during prenatal development (mastoid antrum) but prominent during postnatal growth as air cells enlarge with age and become readily visible, with completion of pneumatization to be around age 10 [6, 15, 27]. Following a thorough literature search, this review is the first scoping review on the size of mastoid air cells with respect to age. Since the first study conducted on the growth of the mastoid air cell system with age conducted by Diamant [41], very few studies have reported the size of mastoid air cells with respect to age involving different age groupings and measurement methods.

Although, a study by Cinamon [8] supported the opinion of Virapongse *et al*. [27] on the development of pneumatization to be three stages from birth till adult size (infantile stage- birth to 2 years; transitional stage- 2 to 5 years; and adult stage- 6 years above), the present review has revealed some differences in the size of mastoid air cells with age. The ontogenetic study by Hill [6] using human cadaveric temporal bone reported air cells to be limited to the mastoid antrum, but area and volumes double before age 4. A twofold to threefold increase in the size of air cells was observed between age 9 and adulthood.

In studies that utilized planimetric measurements (area), a linear pattern of air cell growth was observed among some populations with different ethnic origins from age 1 to 6. However, the growth rate and size of air cells with age still differ. This was observed among Scandinavian and Japanese children, where air cells add about 1–1.2 cm^2^ per year between ages 1–6. However, the size of air cells was reported to increase until puberty among the Scandinavian children reaching adult size ~ 12 cm^2^, with an increase of about 4 cm^2^ every 5 years from age 1–15. There was no report on the size of mastoid air cells after age 6 for Japanese children, and the maximum air cell size reached was 6.68 cm^2^. Among South Asian children (North Indian population and Bangalee people of India), the size of mastoid air cells increases about 2 cm^2^ every 5 years terminating at puberty, reaching an adult size of about 9.66 cm^2^ among the North Indian population. However, there was a continuous increase after puberty reported among the Bangalee people of India, even after age 20 with adult size ~ 16 cm^2^. Isono *et al*. [48] and Lee *et al*. [49] used volumetric measurements on CT images in the East Asian region (Japanese and Korean populations). They reported that the volume of mastoid air cells increased with age. Though both studies were age-related, Isono *et al*. [48] did not specify their age grouping. However, it was observed that the volume of mastoid air cells among the Japanese population increased rapidly until puberty.

Nevertheless, the study of Lee *et al*. [49] specified their age grouping (10-year consecutive age group) and observed among the Korean people that mastoid air cells continue to grow until 30 years with a rapid increase observed among females until puberty with a maximum volume of 7320.6 mm^3^. However, the increase in males steadies till age 29 with a maximum volume of 8211.7 mm^3^. Nonetheless, the highest growth rate reported in this population might be due to the method used.

In addition, from age-related studies that considered sex, the size of mastoid air cells was larger in females until puberty with rapid growth. This could be a reflection of females’ early initiation of general physical growth [8]. However, from the studies that considered ages above puberty, it was reported that the mastoid air cells in males became larger after puberty with a corresponding increase in size and growth rate.

Based on the fact that this review considered studies that measured area and volume air cells, information retrieved suggests that the size of air cells follows a linear growth pattern from age 1 to 6. This is followed by a rapid increase in size from age 6 until puberty and a slow, steady growth from puberty until early adulthood. This, however, contradicts the review and reference study of Cinamon [8], who suggested a slower increment in the size of air cells up to adult size at puberty, but did not elaborate on the growth rate from puberty to early adulthood. Information retrieved from this study also suggests variations in the size of mastoid air cells with age regarding sex and ethnicity.

Furthermore, in ENT and its related surgeries, the interest in the size of mastoid air cells and its importance arose from the association between temporal bone aeration and otitis media either as a cause or a consequence. It may also be considered when planning chronic ear and middle ear surgeries [8]. This is because the mastoid air cell system has been noted as an air reservoir for the middle ear and the volume of air cells governs the capacity of this reservoir [49]. A small mastoid air cell has also been linked to chronic middle ear disease [15]. In human development and growth, the size of mastoid air cells has been well documented to increase with age [2, 4]. Both areas and volumes can measure the size of mastoid air cells. However, volumetric measurements likely give the foremost comprehensive insight to appreciate the estimate of mastoid air cells because it measures three-dimensional space while area gives only surface (2D) information [8]. The present review notes that most of the available information on the mastoid air cell system size with age was based on the surface area through planimetric measurements. However, volumetric estimation of mastoid air cells with age was limited to three studies [6, 48, 49]. Although the study of Lee *et al*. [49] reported the mean volume of air cells in children (0–10 years) to be 3813.85 mm^3^ and adults (19–44 years) to be 7095.20 mm^3^, there is a dearth of information on the volume of air cells in the suggested three stages of development of temporal bone pneumatization owing to the large age grouping employed in their study. Besides, Isono *et al*. [48] and Hill [6] utilized inconsistent age grouping and could not provide pediatric air cell volume information. This means that these studies lack information on the volume of air cells in the infant, transitional, and adult stages. What then is the volume of air cells of healthy temporal bone pneumatization in these developmental stages? Is there any variation in the volume of these air cells with age? Hence, information on the volume of mastoid air cells developmentally remains limited.

## Conclusion

The findings from this review indicate that the size of mastoid air cells increases beyond puberty, even up to 20 years of age among populations of separate ethnic groups. The use of volumetric analysis reported air cells to increase up to around 30 years of age. This review also revealed that the knowledge on the volume of mastoid air cells with respect to age is still lacking despite the comprehensive insights volumetric analysis gives in estimating air cell sizes and their clinical importance. Furthermore, the present study also highlights that no age-related studies on the size of mastoid air cells and temporal bone pneumatization have been conducted in sub-Saharan Africa and low-income countries considering the global prevalence of otitis media and its high incidence rate among children in sub-Saharan Africa. Finally, this review has shown that there is little in the form of research publications on the size of mastoid air cells with age and development of temporal bone pneumatization, as a total of 9 unique age-related articles were found describing this. Further age-related studies are encouraged on the growth and development of the mastoid air cell system and temporal bone pneumatization utilizing volumetric measurement to give a comprehensive insight and a more consolidated description of the size of air cells across age groups in different populations. Three-dimensional reconstructive volumetric analysis is also encouraged to reveal possible morphological (shape) variations of the mastoid air cell system with age.

## Supporting information

S1 Checklist PRISMA checklist. (DOCX)
